# Reducing mental health-related stigma in primary health care settings in low- and middle-income countries: a systematic review

**DOI:** 10.1017/S2045796018000458

**Published:** 2018-09-04

**Authors:** E. Heim, B. A. Kohrt, M. Koschorke, M. Milenova, G. Thornicroft

**Affiliations:** 1 Department of Psychology, University of Zurich, Zurich, Switzerland; 2 Department of Psychiatry, George Washington University, Washington, DC, USA; 3 Centre for Global Mental Health, King's College London, London, UK

**Keywords:** Education psychiatric, mental illness stigma, primary care, systematic reviews

## Abstract

**Aims:**

This systematic review compiled evidence on interventions to reduce mental health-related stigma in primary health care (PHC) in low- and middle-income countries (LMICs). Studies targeting PHC staff (including non-professionals) were included. Primary outcomes were stigmatising attitudes and discriminatory behaviours.

**Methods:**

Data collection included two strategies. First, previous systematic reviews were searched for studies that met the inclusion criteria of the current review. Second, a new search was done, covering the time since the previous reviews, i.e. January 2013 to May 2017. Five search concepts were combined in order to capture relevant literature: stigma, mental health, intervention, PHC staff and LMICs. A qualitative analysis of all included full-texts was done with software MAXQDA. Full-texts were analysed with regards to the content of interventions, didactic methods, mental disorders, cultural adaptation, type of outcome measure and primary outcomes. Furthermore, a risk of bias assessment was undertaken.

**Results:**

A total of 18 studies were included. Risk of bias was rated as high in most included studies. Only six studies had tested their intervention against a control condition, two of which had used random allocation. Most frequently used interventions were lectures providing theoretical information. Many studies also used interactive methods (*N* = 9), discussed case studies (*N* = 8) or used role plays (*N* = 5). Three studies reported that they had used clinical practice and supervision. Results of these studies were mixed. No or little effects were found for brief training interventions (e.g. 1 h to 1 day). Longer training interventions with more sophisticated didactic methods produced statistically significant changes in validated stigma questionnaires. These results have to be interpreted with caution due to risk of bias. Methods for cultural adaptation of interventions were rarely documented.

**Conclusions:**

More rigorous trials are needed in LMICs to test interventions that target discriminatory behaviours in relationship with patients. Cultural adaptation of stigma interventions and structural/institutional factors should be more explicitly addressed in such trials.

## Introduction

In low- and middle-income countries (LMICs), the discrepancy between the high prevalence rates of mental disorders on the one hand, and limited availability of mental health services on the other hand, is well documented (Kessler *et al*., 2007; WHO, 2009). As a response to this ‘treatment gap’ (Saxena *et al*., 2007), the World Health Organisation (WHO) launched the mental health Gap Action Programme (mhGAP, WHO, 2008) which aims at scaling up services for mental, neurological and substance use disorders, particularly in LMICs. Integrating mental health into primary health care (PHC) is a key component of the mhGAP programme. By integrating mental health into PHC, people in need of treatment can access services easily and be transferred to specialised care if necessary (Funk *et al*., 2008). Additionally, the mhGAP programme supports training community health workers in taking on limited tasks in the care of people with mental illness, such as case detection, referral to PHC and providing psychosocial support (Keynejad *et al*., 2018).

This *task-sharing* approach (Patel, 2009), in which care of people with mental illness is transferred from specialists to also involve primary care and community health workers, has been increasingly implemented in many LMICs over the past decade. WHO has developed an Intervention Guide (IG) for mental, neurological and substance use disorders in non-specialised health settings (WHO, 2016) in order to support this task-sharing process. A recent systematic review compiled evidence on the practical implementation of the WHO mhGAP-IG in LMICs and showed promising results (Keynejad *et al*., 2018). Despite these efforts to enhance provision of mental health care in LMICs, and increasing evidence showing their positive results, many people with mental illness in LMICs still do not receive adequate treatment.

Mental health-related stigma is a key barrier to mental health care (Saxena *et al*., 2007). ‘Stigma’ can be conceptualised in terms of knowledge (i.e. ignorance or misinformation), attitude (i.e. prejudice) and behaviour (i.e. discrimination, violence, hostility and human rights abuses) (Thornicroft, 2006). A systematic review revealed stigmatising beliefs, attitudes and discriminatory behaviours among primary and specialised health care professionals in both high-income countries (HICs) and LMICs (Henderson *et al*., 2014). Thus, even if people with mental illness overcome barriers and seek help in PHC, they are still at risk of being confronted with stigmatising beliefs, negative attitudes and discrimination, which hinders their right to adequate care.

Empirical evidence on how to reduce stigma in specific populations (e.g. health care workers) is generally scarce, as most studies merely *assess* stigma but do not test interventions to actually *address* it (Evans-Lacko *et al*., 2014). Studies in the general population showed that interventions containing social contact and first-person narratives were more effective than others (Mehta *et al*., 2015), a finding that could not be confirmed for health professionals (Henderson *et al*., 2014). Evidence from LMICs is particularly limited (Henderson *et al*., 2014; Mehta *et al*., 2015), especially when it comes to evidence on effective interventions, on how best to target key groups such as healthcare staff, or on how to culturally adapt interventions to local contexts (Semrau *et al*., 2015).

Stigma can be addressed in specific interventions with PHC staff, but ideally it is incorporated into training as an integral part of health and mental health education. In LMICs, in the context of implementing the mhGAP, professional health staff in primary care as well as lay community mental health workers is increasingly trained in the detection and evidence-based treatment of mental disorders. Stigma would ideally be integrated into these training sessions, but evidence is still lacking on how to address stigma in these settings. In a systematic review, Mehta *et al*. (2015) reviewed interventions to reduce mental health-related stigma among different populations. This review revealed three interventions targeting health professionals in LMICs, hence evidence is limited.

Previous systematic reviews also showed that outcome assessments to quantify the effectiveness of stigma interventions remain an empirical challenge. While knowledge and attitudes can be measured using questionnaires, discriminatory behaviours are more difficult to assess. In the systematic review by Henderson *et al*. (2014) among health care staff, little evidence was found on behavioural outcomes of stigma interventions, and none of these studies were conducted in LMICs. This lack of evidence might be explained by the fact that behavioural outcomes are ideally measured by asking patients about their experiences with health professionals, and such data are more difficult to collect than assessments of knowledge and attitudes. And finally, stigma is most likely shaped by culture (Yang *et al*., 2007; Yang *et al*., 2014). Systematic reviews show that cultural adaptation of psychological interventions increases their effectiveness (e.g. Harper Shehadeh *et al*., 2016), but this has not yet been tested for stigma interventions.

In summary, it is most relevant to better understand how mental health-related stigma can be addressed in trainings with health workers in the context of task-sharing approaches in LMICs. Previous reviews have not answered this question. The current study aims to close this gap by providing evidence on interventions for reducing mental health-related stigma among PHC workers in LMICs, with a primary focus on attitudes and behaviours. It therefore covers a small intersection of studies that were included in previous reviews (Henderson *et al*., 2014; Mehta *et al*., 2015; Semrau *et al*., 2015). A new search was run to also include studies that have been published since these previous reviews. Furthermore, by using a ‘magnifying glass strategy’, a closer look is taken on the content and didactic methods used in stigma interventions for PHC staff in LMICs, and on the cultural adaptation of such interventions. With this approach, the current review aims to highlight future directions for designing effective interventions, thereby contributing to enhancing the quality of mental health care in primary care settings in LMICs.

## Data collection, extraction and analysis

**Fig. 1.**
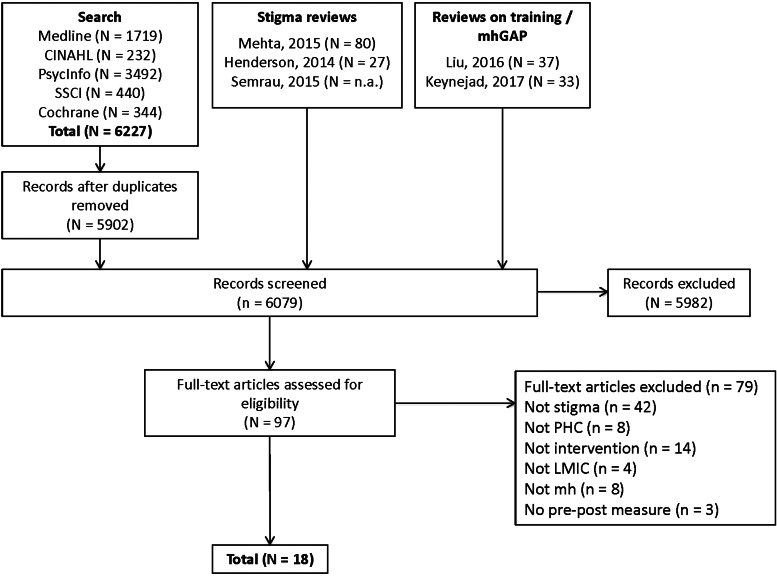
Preferred Reporting Items for Systematic Reviews and Meta-Analyses (PRISMA) diagram with a systematic search and selection process.

This study was listed in the PROSPERO register for systematic reviews (registration number CRD42017065436). Data collection included two different strategies. First, the existing systematic reviews on either stigma among PHC staff (Henderson *et al*., 2014; Mehta *et al*., 2015; Semrau *et al*., 2015) or training of PHC staff in LMICs (Liu *et al*., 2016; Keynejad *et al*., 2018) were searched for studies that met the inclusion criteria of the current review. Second, a new search was done, covering the time since the previous reviews, i.e. January 2013 to May 2017. The new search was run on 6 May 2017 and covered the following databases: PsycINFO, MEDLINE (Ovid), CINAHL, Social Sciences Citation Index and Cochrane (only Trials). Five search concepts were combined in order to capture relevant literature: stigma (e.g. stigma, discrimination or stereotype); mental health (e.g. depression, anxiety or schizophrenia); intervention (e.g. randomised controlled trial, evaluation or pre-post); PHC staff (e.g. general practitioners or health care workers) and LMICs classified according to the World Bank (2016), using their names (e.g. Afghanistan) and population adjectives (e.g. Afghan). The search strategies were adapted from the previous reviews. The complete search strategy (only Medline) can be accessed in the online Supplementary Material. The PRISMA diagram showing the data collection process is given in Fig. 1.

Inclusion and exclusion criteria were defined along the participants, interventions, comparators and outcomes (PICO) approach. Participants in stigma interventions were PHC staff in LMICs (including non-professionals). The intervention had to be a mental health-related training programme addressing PHC workers’ knowledge, attitudes and behaviours in terms of professional skills. Studies were excluded if they trained or evaluated knowledge and behaviour/skills only, without an attitudinal (stigma-related) component, or if they did not include a pre-training assessment. Accordingly, outcomes had to include an attitudinal assessment, aside from knowledge and/or skills. Both qualitative and quantitative studies were included, and no comparator was defined.

The included full-texts were introduced to the qualitative data analysis software MAXQDA (version 12.3.3). A coding system was developed including the following categories: stigma intervention content (e.g. theory, diagnostic skills, relationship skills), didactic methods (e.g. lecture, role plays, contact with patients), the mental disorder the intervention focused on (e.g. depression, psychosis), whether the intervention was culturally adapted, the type of outcome measure (e.g. validated or non-validated questionnaire, qualitative interviews, behavioural observation) and primary outcomes. The analysis of outcomes focused only on attitudes and behaviour, not on knowledge. Outcome measures regarding knowledge are very diverse and would provide results that are hardly comparable, and knowledge was not the main focus of the current review. Two raters coded the full-texts independently and discussed possible differences until finding an agreement. Additionally, we used a category system to critically appraise the methodological quality of the included studies considering the following five criteria: control group, random allocation, random sequence generation, allocation concealment and incomplete data. A meta-analysis was originally planned but could not be calculated for the reasons given below.

## Results

Eighteen studies were included in the analysis (see Table 1). The studies were from 11 different countries and included a broad range of participants, such as nurses, general practitioners (GPs) or community mental health workers. Sample sizes ranged from lower than *N*  =  30 to *N* > 200, with seven studies having sample sizes of *N* > 100. Most studies (*N*  =  11) covered mental disorders in general, but some studies addressed one particular disorder, such as depression (*N*  =  4) and schizophrenia (*N*  =  2).

**Table 1.** Summary of included studies describing training with PHC workers

Reference	Country	Target population	*N*	Intervention: content (duration)	Design	Measure(s)	Main results (descriptive analysis)
Abayomi *et al*. (2013)	Nigeria	Volunteers for the provision of mental health services	31	Educational sessions based on a mental health literacy manual (six sessions, 1 h each)	Pre-post	Modified Bogardus Social Distance Scale (Adewuya and Makanjuola, 2008)	Statistically significant decrease in perceived dangerousness of people with mental illness (*p* *<* 0.01) and social distance (*p* = 0.002).
Alexander *et al*. (2013)	Kenya	Nurses	23	Training on depression screening (1 h)	Waitlist control (randomised allocation)	DAQ (Botega *et al*., 1992)	No difference in participants’ professional ease with depressed patients as measured by the DAQ was observed between the intervention and control group (means and standard deviations were reported, but no result of statistical test).
Armstrong *et al*. (2011)	India	Community mental health workers	70	Introduction to mental health and mental disorders, mental health first aid, practice-based skills and mental health promotion (4 days)	Pre-post	Two vignettes (depression and psychosis), assessment of attitudes based on seven statements (binary response format)	– Statistically significant changes in two statements on depression between the baseline and 3-month follow-up (‘sign of personal weakness’ from 84.8 to 62.1%; ‘social distance’ from 21.2 to 4.5%). – No statistically significant changes in responses to statements regarding psychosis between the baseline and 3-month follow-up (significant changes occurred in three statements between the baseline and post-assessment but were not sustained).
Bradshaw *et al*. (2006)	South Africa	Voluntary health workers (*N* = 21)	21	Training on understanding mental illness, how to intervene and how to support families and communities (18 h)	None	Qualitative assessment (focus groups pre- and post-training)	Reduction of people agreeing that aggressive behaviour is a sign of mental illness.
Chinnayya *et al*. (1990)	India	Primary care paramedical health workers	150	Short-term training in mental health care (1 week)	Pre-post	Attitude Questionnaire, including 35 items to assess attitudes towards psychosis, epilepsy and mental retardation (binary response format)	– Significant changes in percentage of positive responses to 25 items from pre- to post-assessment. – Statistically significant change in the total number of positive answers from pre- to post-assessment (*p* < 0.001).
Cui *et al*. (2015)	China	Rural specialists (candidate general practitioners)	198	Mental health training programme to improve specialists’ knowledge and attitudes (6 weeks, 8 h a week)	Pre-post	Attitudes Questionnaire developed by the Ministry of Public Health of China (no further information on content or response format published)	Candidate general practitioners' attitudes towards people with mental health problems became more positive (no statistical parameters published).
da Silva Cais *et al*. (2011)	Brazil	Health professionals, mainly primary care workers	135	Suicide prevention training (18 h)	Pre-post	SBAQ (Botega *et al*., 2005), 21 items (response format: analogical visual scale from 0 to 10)	Statistically significant change in 18 single items and in the three SBAQ subscales, i.e. negative feelings in relation to patients with suicidal behaviour (*p* = 0.002), suicide rights (*p* = 0.02), and professional capacity perception (*p* < 0.001).
Hofmann-Broussard *et al*. (2017)	India	Community health workers	56	Twelve-module course including contact intervention (4 days)	Controlled pre- and post-design. Comparator: no training	Stigma Questionnaire (Jadhav *et al*., 2007), based on two vignettes (depression and psychosis)	– Statistically significant reduction of stigmatising attitudes towards people with depression within intervention group from pre- to post (*p* = 0.005), but not between the groups at follow-up (*p* = 0.418). – Statistically significant reduction of stigmatising attitudes towards people with psychosis within intervention group from pre- to post (*p* < 0.001) and between the groups at follow-up (*p* = 0.016).
Li *et al*. (2014a)	China	Community mental health staff	99	Training to increase knowledge and reduce stigma (1 day)	Pre-post	– RIBS (Evans-Lacko *et al*., 2011), Chinese version (Li *et al*., 2014b) – MICA (Kassam *et al*., 2010)	– The mean score of MICA significantly decreased between the pre- and post-assessment (*p* < 0.001, Cohen's *d* = 0.48). – The mean score of RIBS significantly increased between the pre- and post-assessment (*p* < 0.001, Cohen's *d* = 0.34).
Li *et al*. (2015)	China	Community mental health staff	77	Mental health training from a public health perspective (14 days)	Cluster randomisation (districts), pre-, post- and follow-up. Comparison between the new and old curriculum.	– MAKS (Evans-Lacko *et al*., 2010), – RIBS (Evans-Lacko *et al*., 2011), Chinese version (Li *et al*., 2014b), – MICA (Kassam *et al*., 2010)	– MAKS: no significant group × time interaction. – MICA: significant group × time interaction at 6-month follow-up (*p* < 0.001). – RIBS: significant group × time interaction at 6- and 12-month follow-up (*p* < 0.001).
Makanjuola *et al*. (2012)	Nigeria	Training of trainers for primary care providers	24	Workshop for trainers in the use of a structured package of mental health training materials (1 week)	Pre-post	Thirteen statements on knowledge and attitudes (binary response format)	Significant change in responses to five items, all of which address knowledge. No significant change in questions regarding attitudes (e.g. people with mental illness are dangerous).
Motallebi (1996), included in systematic review by Mansouri *et al*. (2009)	Iran	*Behvarzes* (local health workers) (*N* = unknown)		Unknown	Unknown	Unknown	Significant changes attitudes (*p* < 0.01).
Sadik *et al*. (2011)	Iraq	Health workers in primary care centres, i.e. general practitioners, healthcare workers, nurses, social workers and others	317	Training toolkit developed for Kenya and used in other countries (2 weeks)	Controlled pre-post design. Comparator: no training	– Knowledge, attitudes and practice questionnaire – Direct observation of health workers' skills with regards to the contact with patients – Exit interviews with patients after the consultation	– Improved test scores on the questionnaire from a pre-training average score of 42.3 to a post-training score of 59%. – A greater proportion of the trained than the untrained physicians displayed excellent skills (no statistical test). – A higher proportion of patients of trained physicians reported overall satisfaction with the care received than did patients of non-trained physicians (85.6% *v*. 69.3%, *p* < 0.001).
Shirazi *et al*. (2009)	Iran	General practitioners	192	Training on the diagnosis and treatment of depression (2 days)	Randomised controlled trial, comparing conventional training *v*. interactive educational methods	DAQ (Botega *et al*., 1992)	The attitude statements improved significantly in both study arms but no differences were found between the intervention and the control arm (data not published).
Ucok *et al*. (2006)	Turkey	General practitioners	54	Stigma intervention addressing attitudes towards schizophrenia (one session)	Pre-post	Thirteen-item questionnaire focussing on doctors’ views and attitudes towards schizophrenia (binary response format)	Statistically significant, positive changes on five items, including items about the treatability of schizophrenia, harmfulness and untrustworthiness of schizophrenic patients.
Vesel *et al*. (2015)	Sierra Leone	Health workers	271	Stress-management intervention (duration not specified)	Pre-post, control design compared with neighbour district	– Self-reported relationship with patients (questionnaire) – Qualitative interviews	– Improved self-reported relationship with patients (the survey was administered after the intervention to assess both the pre- and post-assessment). – Qualitative: change in health workers’ behaviours and attitudes towards their clients after the counselling and training they received.
Villamil-Salcedo *et al*. (2017)	Mexico	General practitioners	n.a.	Collaborative care model (duration not specified)	Qualitative assessment	Questions on stigma	– After the Collaborative Care model was applied, general practitioners were more aware about mental health problems and they were more interested in the identification of these conditions. – Regarding the questions of stigma, 29% of them reported being afraid of treating patients with mental disorders; 24% reported that they get easily angry when they treat a person with a mental disorder.
Wang *et al*. (2012)	China (Taiwan)	Non-psychiatric physicians	307	Postgraduate educational programme on depression (2 days)	Pre-post	– DAQ (Botega *et al*., 1992)	More positive attitudes towards depression (*p* < 0.001 on three subscales and *p* = 0.002 on one subscale), with effect sizes (Hedge's *g*) between 0.19 and 0.44 on the four subscales.

*Note:* DAQ, Depression Attitude Questionnaire; SBAQ, Suicide Behaviour Attitude Questionnaire; VAS, Violence Attitude Scale; RIBS, Reported and Intended Behaviour Scale; MICA, Mental Illness: Clinicians’ Attitudes Scale; MAKS, Mental Health Knowledge Schedule.

The majority of interventions (*N*  =  15) provided theoretical information, such as symptoms, prevalence and aetiology of mental disorders, relationship between the mental and physical health or social consequences of mental disorders. In seven studies, stigma was explicitly addressed in this theoretical introduction. As an example, Ucok *et al*. (2006) described their intervention as follows: ‘the slide presentation lasted approximately 45 minutes and included current information on the course of schizophrenia and its treatment, the impact of stigma on schizophrenia, and description of GPs’ roles’ (p. 440).

The majority of the studies (*N*  =  11) covered the treatment of mental disorders, describing a variety of interventions, such as treatment guidelines, medication, counselling, referral to specialists, psychological first aid, problem-solving or interpersonal therapy. Of those studies, four reported that they had explicitly addressed the relationship with patients, e.g. promoting communication skills. As an example, Bradshaw *et al*. (2006) described that their community volunteers’ education programme included ‘examining interactions which may increase or reduce stress for the patient’ (p. 100). A great number of studies (*N*  =  11) aimed at improving participants’ diagnostic skills. Some interventions covered mental health policy (*N*  =  3), mental health promotion in communities and psychosocial interventions (*N*  =  3) and stress management (*N*  =  2).

With regards to didactic methods, the vast majority of studies provided lectures (*N*  =  12). Additionally, many studies used interactive methods (*N*  =  9), discussed case studies (*N*  =  8) and used role plays (*N*  =  5). Some interventions handed out written material (*N*  =  5) or used multimedia as a didactic method (*N*  =  3). Only three studies reported that they had used clinical practice and supervision as an intervention, and in one study, a patient told his recovery story to participants. The amount of information provided on the content and didactic methods used in interventions varied greatly. One study published the intervention manual online (Armstrong *et al*., 2011), whereas other studies provided only minimal information.

Four studies reported that they had adapted their interventions or measures to the specific cultural context, but the descriptions remained rather vague. As an example, Bradshaw *et al*. (2006) reported that ‘attempts were made to ensure that course materials were culturally appropriate to a South African community’ (p. 99). Outcomes of these interventions were most often measured using non-validated questionnaires (*N*  =  11) or validated questionnaires (*N*  =  6). Additionally, two studies used qualitative interviews for measuring outcomes, one study used interviews with patients and direct observation of health workers' skills, and two studies measured behavioural intentions.

With regards to risk of bias, the quality of the included studies varied greatly, but no study showed low risk of bias (the full risk of bias assessment is available in the online Supplementary Material). Only six studies compared their intervention with a control condition (Shirazi *et al*., 2009; Sadik *et al*., 2011; Alexander *et al*., 2013; Li *et al*., 2015; Vesel *et al*., 2015; Hofmann-Broussard *et al*., 2017), two of which used random allocation. Four studies applied quasi-experimental designs, e.g. testing an intervention in one district and using another district as a control group. All studies had administered self-reported outcome measures; thus, no blinding of outcome assessment was done. Many studies reported high drop-out rates, but only one study used intention-to-treat analysis for dealing with missing data. No meta-analysis could be calculated, for the following reasons: of the six studies that had compared their intervention with a control condition, two studies published no or incomplete data (Shirazi *et al*., 2009; Alexander *et al*., 2013), two studies presented outcome measures that could not be used in meta-analysis (Sadik *et al*., 2011; Vesel *et al*., 2015) and one study compared two different didactic methods (Li *et al*., 2015).

The descriptive analysis of the outcomes is displayed in Table 1. Most studies found some kind of positive effects of their intervention on attitudes of PHC staff towards people with mental illness, but some of these effects were rather small, e.g. statistically significant change in the percentage of positive responses to single items with binary response format (Chinnayya *et al*., 1990; Ucok *et al*., 2006; Armstrong *et al*., 2011). Some studies reported positive outcomes but did not present results of their statistical analyses (Shirazi *et al*., 2009; Cui *et al*., 2015). No or little effects were found from short training interventions, e.g. a 1-h training on depression screening (Alexander *et al*., 2013) or a one-session stigma intervention for general practitioners (Ucok *et al*., 2006).

Differences regarding types of mental disorders emerged, although inconsistently. Armstrong *et al*. (2011) found a change in attitudes towards people with depression but not towards people with psychosis, whereas Hofmann-Broussard *et al*. (2017) found a stronger effect on attitudes towards psychosis than depression. Two studies compared different curriculums or didactic methods (Shirazi *et al*., 2009; Li *et al*., 2015), but only one found significant group differences (Li *et al*., 2015). Several studies showed a statistically significant change in total scores or subscales of questionnaires (da Silva Cais *et al*., 2011; Wang *et al*., 2012; Li *et al*., 2014*a*; Li *et al*., 2015; Hofmann-Broussard *et al*., 2017), but these results must be interpreted with caution due to risk of bias.

Few studies assessed behavioural outcomes alongside attitudes. In one study (Sadik *et al*., 2011), psychiatrists evaluated the job skills of trained and untrained PHC staff. Evaluators were blinded to participants’ group assignment. In addition, patients were interviewed. The study showed differences between the study groups. And two studies (Li *et al*., 2014*a*; Li *et al*., 2015) applied the Reported and Intended Behaviour Scale (Evans-Lacko *et al*., 2011) to assess behavioural discrimination. Li *et al*. (2015) found a significant group × time interaction, indicating a difference between the two didactic methods tested in the study (see Table 1).

## Discussion

This systematic review looked at interventions to reduce stigma among a variety of PHC workers (e.g. general practitioners, nurses, community health workers or volunteers) in LMICs. A total of 18 studies were included. The quality of included studies varied greatly, with a high number of studies showing a high risk of bias. Six studies (33%) have tested their intervention against a control condition, and only two studies (11%) had used random allocation. Moreover, a large number of studies reported high numbers of incomplete data but did not provide any information on how they dealt with missing values. From the present state of the literature, no meaningful conclusions can be drawn on the effectiveness of stigma interventions, key ingredients or target populations within health care. In the following, we aim to highlight suggestions on how to design future studies, in order to enhance the amount and the quality of evidence.

As reported in a previous systematic review on stigma interventions with PHC staff (Henderson *et al*., 2014), all included studies measured outcomes by assessing knowledge and attitudes, with only a few studies measuring behavioural outcomes. Accordingly, providing theoretical information through lectures was the most frequent intervention, and more practical interventions targeting discriminatory behaviours, such as role plays or clinical practice under supervision, were rarely used. Studies testing such practical interventions are needed to target communication skills and relationship with patients. The protocol for such a study has been published recently (Kohrt *et al*., 2018).

Types of attitudinal outcome measures varied greatly, ranging from single items with binary response format to validated stigma questionnaires. Items with binary response format do not allow for more complex statistical procedures such as principal component analysis, inferential statistics, multiple imputation of missing values or inclusion of the results in meta-analyses. Moreover, the large diversity of questionnaires used across studies makes it difficult to obtain results, since the underlying constructs of these measures may vary. Consensus is needed on how to measure stigma in order to assess the efficiency of stigma interventions in the future.

Having said that, such a consensus should be inclusive for cross-cultural variations in how stigma is assessed and how interventions are designed (Yang *et al*., 2014). Several studies reported cultural adaptation of questionnaires, but without using standardised methods for ensuring their validity and reliability, e.g. measurement invariance testing (e.g. Byrne, 2008) or cognitive interviewing. Furthermore, few of the included studies have made an attempt to culturally adapt their interventions. The importance of culturally adapting psychotherapeutic interventions has increasingly been stressed (Chowdhary *et al*., 2014). It is most likely that stigma interventions require similar procedures for cultural adaptation, since how stigma is experienced varies across cultures (Yang *et al*., 2007; Yang *et al*., 2014). Aside from culture, structural and institutional factors may affect how stigma materialises in PHC, e.g. whether a country has a mental health policy, the amount of financial and human resources allocated for mental health, how the mental health system is composed and the level of training of health workers. Taking into account such structural and institutional factors in future studies would enhance their comparability.

## Limitations

This review has several limitations. First, we relied on previous reviews for the time before 2013. Second, included studies were published in English, with one exception. Full-text screening in other languages was done where necessary, but no specific search engines such as Scielo were used, and we did not include grey literature. Third, we did not look at outcomes of the interventions in terms of knowledge. Measuring knowledge in the field of mental health is a broad topic, and summarising this evidence would have been beyond the scope of the current review, due to its main focus on attitudes and behaviour.

## Conclusions

This systematic review provides pathways for future research in stigma interventions for PHC staff. More practical interventions should be implemented and tested in LMICs using more rigorous methods with regards to research design, outcome measures, statistical analysis and dealing with missing data. Moreover, consensus is needed on how to measure stigma, alongside cultural adaptation of both assessment instruments and interventions. The global mental health ‘treatment gap’ and the integration of mental health into PHC requires evidence-based interventions for addressing stigma, in order to increase access to treatment and provide high-quality care to people suffering from mental disorders worldwide.

## Supple>Supplementary material

For supplementary material accompanying this paper visit http://dx.doi.org/10.1017/S2045796018000458. click here to view supplementary material

## Availability of data and materials

The data extraction spread sheet (Excel) is available on request, please contact the corresponding author.
