# Supplementary material for: Reducing mental health-related stigma in primary health care settings in low- and middle-income countries: a systematic review
Source: Epidemiol Psychiatr Sci. 2018 Sep 4;29:e3. doi: 10.1017/S2045796018000458 (PMC6399081; doi:10.1017/S2045796018000458)
Supplement: Supplementary file 1 [file S2045796018000458sup001.pdf]

MEDLINE SEARCH STRATEGY
2017-05-06

1. Stigma (Mehta et al., 2015; Thornicroft et al., 2016)
Stereotyping/ OR Social Stigma/ OR Prejudice/ OR Attitude/ OR Social Perception/ OR "Attitude of Health Personnel"/ OR Social Distance/ OR "Rejection (Psychology)"/ OR Human Rights/ OR Civil Rights/ OR Social Justice/ OR "Discrimination (Psychology) "/ OR Social discrimination/ OR human rights OR civil rights OR social justice OR discrimination OR injustice OR stigma* OR stereotyp* OR social distanc* OR marginali*

Additional terms by Henderson et al. (2014): prejudi* OR Ignoran* OR Misconception*1 OR Misperception*1 OR hostil* OR emotional reaction OR disrespect* OR victimi* OR abusive OR patronis* OR patroniz* OR misdiagnos* OR under treatment OR non treatment OR no access OR less access OR diagnostic overshadow* OR attitud* OR behavior*1 OR behaviour*1 OR opinion*1 OR view* OR assum* OR judg*

Additional terms by authors: Professional Competence/ OR Quality of Health Care/ OR Patient Satisfaction/ OR Patient Acceptance of Health Care/ OR Burnout, Professional/ OR case detection OR competen* OR practice OR skills OR burnout OR dignity OR help-seeking

Exclude (from Mehta et al., 2015; Thornicroft et al., 2016)
6. Interpersonal Relations/
7. public opinion/
14. public opinion
19. empower*

2. Mental health (Mehta et al., 2015; Thornicroft et al., 2016)
Mental Health/ OR Mental Disorders/ OR Mentally Ill Persons/ OR Somatoform Disorders/ OR Depression/ OR Depression, Postpartum/ OR common mental disorders OR Depressive Disorder/ OR Depressive Disorder, Major/ OR Psychotic Disorders/ or Affective Disorders, Psychotic/ OR Neurotic Disorders/ or Self-Injurious Behavior/ OR Anxiety/ OR Anxiety Disorders/ OR Schizophrenia, Paranoid/ OR Schizophrenia/ OR Bipolar Disorder/ OR Mood Disorders/ OR Obsessive-Compulsive Disorder/ OR Personality Disorders/ OR Eating Disorders/ OR Anorexia Nervosa/ OR Anorexia/ OR Bulimia Nervosa/ or Bulimia/ OR Mental Health Services/ OR mental health OR mental illness OR psychotic OR psychosis OR schizo* OR bipolar disorder OR manic depression OR OCD OR obsessive compulsive disorder OR anorex* OR bulimi*

Additional terms by Henderson et al. (2014): post-traumatic stress OR alcohol* OR substance OR illicit drugs

Additional terms by authors: OR PTSD OR distress

3. Intervention (Mehta et al., 2015; Thornicroft et al., 2016)
randomized controlled trial.pt. OR controlled clinical trial.pt. OR evaluation studies.pt. OR Prospective Studies/ OR Follow-Up Studies/ OR Social Marketing/ OR National Health Programs/ OR Government Programs/ OR Program Development/ OR Learning/ or Multimedia/ or Teaching/ OR Internet/ OR Video-Audio Media/ OR Video Recording/ OR Software/ OR Persuasive Communication/ OR Health Knowledge, Attitudes, Practice/ OR Awareness/ OR Patient Advocacy/ OR Health Promotion/ OR Intervention Studies/ OR Randomized Controlled Trials as Topic/

OR evaluat* OR intervention* OR randomi* OR follow-up OR pre-post OR time series OR time point OR message OR marketing OR program* OR newsletter* OR DVD OR website OR compact disc OR stigma resilience OR stigma change OR (stigma* adj5 cop*) OR (stigma* adj5 reduc*) OR

(discrim* adj5 reduc*) OR (stigma* adj5 challeng*) OR (discrim* adj5 challeng*) OR (stigma* adj5 combat*) OR (discrim* adj5 combat*) OR (stigma* adj5 counter*) OR (discrim* adj5 counter*) OR contact OR testimon* OR mental health literacy OR training

(excluded)
66. Mass Media/
69. Journalism
70. Video Games/
72. Pamphlets/
73. Advertising as Topic/
74. Public Relations/
75. Public Relations/
77. Famous Persons/
80. Consumer Advocacy/
91. campaign
93. advert*
95. public relation*
97. newspaper*
98. magazine*
100. broadcast
101. radio
102. television
103. cinema
107. movie
108. film
109. trailer

4. Health care (Henderson et al., 2014)

health* professional OR health* personnel OR Doctor*1 OR psychiat* OR clinical psych* OR nurs* OR occupational therap* OR medical student*1 OR health* worker*1 OR paramedic* OR ambulance service OR mental health social worker*1 OR Medic*1 OR Clinician*1 OR Physician*1 OR case manager*1 OR key worker*1 OR keyworker*1 OR psychotherapist*1 OR hospital* OR a&e OR Emergency department*1 OR Health* provider*1 OR Health* staff OR general practitioner*1

Additional terms by authors: Health personnel/ OR Professional-Patient Relations/ OR psychology students

(excluded)
midwi*
physiotherap*
Pharmacist*1
Pharmacy student*1
dietitian*1
dietician*1
Dentist*1
Surgeon*1
Obstetrician*1
Dental hygienist*1
Gynaecologist*

5. LMIC country list

Afghanistan.mp OR Albania.mp OR Algeria.mp OR American Samoa.mp OR Angola.mp OR Argentina.mp OR Armenia.mp OR Azerbaijan.mp OR Bangladesh.mp OR Belarus.mp OR Belize.mp OR Benin.mp OR Bhutan.mp OR Bolivia.mp OR Bosnia OR Herzegovina OR Botswana.mp OR Brazil.mp OR Bulgaria.mp OR "Burkina Faso".mp OR Burundi.mp OR "Cape Verde".mp OR Cambodia.mp OR Cameroon.mp OR "Central African Republic".mp OR Chad.mp OR China.mp OR Colombia.mp OR Comoros.mp OR Congo.mp OR Costa Rica.mp OR "Cote d'Ivoire".mp OR Cuba.mp OR Djibouti.mp OR Dominica.mp OR Dominican Republic.mp OR Ecuador.mp OR Egypt.mp OR "El Salvador".mp OR "Equatorial Guinea".mp OR Eritrea.mp OR Ethiopia.mp OR Fiji.mp OR Gabon.mp OR Gambia.mp OR Georgia.mp OR Ghana.mp OR Grenada.mp OR Guatemala.mp OR Guinea.mp OR Guinea-Bissau.mp OR Guyana.mp OR Haiti.mp OR Honduras.mp OR India.mp OR Indonesia.mp OR Iran.mp OR Iraq.mp OR Jamaica.mp OR Jordan.mp OR Kazakhstan.mp OR Kenya.mp OR Kiribati.mp OR Korea.mp OR Kosovo.mp OR Kyrgyzstan.mp OR Laos.mp OR Lebanon.mp OR Lesotho.mp OR Liberia.mp OR Libya.mp OR Macedonia.mp OR Madagascar.mp OR Malawi.mp OR Malaysia.mp OR Maldives.mp OR Mali.mp OR "Marshall Islands".mp. OR Mauritania.mp OR Mauritius.mp OR Mexico.mp OR Micronesia.mp OR Moldova.mp OR Mongolia.mp OR Montenegro.mp OR Morocco.mp OR Mozambique.mp OR Myanmar.mp OR Namibia.mp OR Nepal.mp OR Nicaragua.mp OR Niger.mp OR Nigeria.mp OR Pakistan.mp OR Palau.mp OR Panama.mp OR "Papua New Guinea".mp OR Paraguay.mp OR Peru.mp OR Philippines.mp OR Romania.mp OR Russia.mp OR Rwanda.mp OR Samoa.mp OR "Sao Tome and Principe".mp OR Senegal.mp OR Serbia.mp OR "Sierra Leone".mp OR Melanesia.mp OR Somalia.mp OR "South Africa".mp OR "South Sudan".mp OR "Sri Lanka".mp OR "Saint Lucia".mp OR "Saint Vincent and the Grenadines".mp OR Sudan.mp OR Suriname.mp OR Swaziland.mp OR Syria.mp OR Tajikistan.mp OR Tanzania.mp OR Thailand.mp OR Timor-Leste.mp OR Togo.mp OR Tonga.mp OR Tunisia.mp OR Turkey.mp OR Turkmenistan.mp OR Tuvalu.mp OR Uganda.mp OR Ukraine.mp OR Uzbekistan.mp OR Vanuatu.mp OR Venezuela.mp OR Vietnam.mp OR "West Bank".mp OR Gaza.mp OR Palestine.mp OR Yemen.mp OR Zambia.mp OR Zimbabwe.mp

Afghanistan/ OR Albania/ OR Algeria/ OR American Samoa/ OR Angola/ OR Argentina/ OR Armenia/ OR Azerbaijan/ OR Bangladesh/ OR Belarus/ OR Belize/ OR Benin/ OR Bhutan/ OR Bolivia/ OR "Bosnia and Herzegovina"/ OR Botswana/ OR Brazil/ OR Bulgaria/ OR "Burkina Faso"/ OR Burundi/ OR Cape Verde/ OR Cambodia/ OR Cameroon/ OR Central African Republic/ OR Chad/ OR China/ OR Colombia/ OR Comoros/ OR Congo/ OR Costa Rica/ OR Cote d'Ivoire/ OR Cuba/ OR Djibouti/ OR Dominica/ OR Dominican Republic/ OR Ecuador/ OR Egypt/ OR El Salvador/ OR Equatorial Guinea/ OR Eritrea/ OR Ethiopia/ OR Fiji/ OR Gabon/ OR Gambia/ OR Georgia/ OR Ghana/ OR Grenada/ OR Guatemala/ OR Guinea/ OR Guinea-Bissau/ OR Guyana/ OR Haiti/ OR Honduras/ OR India/ OR Indonesia/ OR Iran/ OR Iraq/ OR Jamaica/ OR Jordan/ OR Kazakhstan/ OR Kenya/ OR Kiribati/ OR "Democratic People's Republic of Korea"/ OR Kosovo/ OR Kyrgyzstan/ OR Laos/ OR Lebanon/ OR Lesotho/ OR Liberia/ OR Libya/ OR "Macedonia (Republic)"/ OR Madagascar/ OR Malawi/ OR Malaysia/ OR Maldives/ OR Mali/ OR Marshall Islands.mp. OR Mauritania/ OR Mauritius/ OR Mexico/ OR Micronesia/ OR Moldova/ OR Mongolia/ OR Montenegro/ OR Morocco/ OR Mozambique/ OR Myanmar/ OR Namibia/ OR Nepal/ OR Nicaragua/ OR Niger/ OR Nigeria/ OR Pakistan/ OR Palau/ OR Panama/ OR Papua New Guinea/ OR Paraguay/ OR Peru/ OR Philippines/ OR Romania/ OR Russia/ OR Rwanda/ OR Samoa/ OR (Sao Tome and Principe).mp OR Senegal/ OR Serbia/ OR Sierra Leone/ OR Melanesia/ OR Somalia/ OR South Africa/ OR South Sudan/ OR Sri Lanka/ OR Saint Lucia/ OR "Saint Vincent and the Grenadines"/ OR Sudan/ OR Suriname/ OR Swaziland/ OR Syria/ OR Tajikistan/ OR Tanzania/ OR Thailand/ OR Timor-Leste/ OR Togo/ OR Tonga/ OR Tunisia/ OR Turkey/ OR Turkmenistan/ OR Tuvalu.mp OR Uganda/ OR Ukraine/ OR Uzbekistan/ OR Vanuatu/ OR Venezuela/ OR Vietnam/ OR West Bank.mp OR Gaza.mp OR Palestine.mp OR Yemen/ OR Zambia/ OR Zimbabwe/

[retrieved from http://data.worldbank.org/income-level/low-and-middle-income on 24 March 2017]

References

Henderson, C., Noblett, J., Parke, H., Clement, S., Caffrey, A., Gale-Grant, O., . . . Thornicroft, G. (2014). Mental health-related stigma in health care and mental health-care settings. *The Lancet Psychiatry, 1*(6), 467-482. doi:10.1016/S2215-0366(14)00023-6

Mehta, N., Clement, S., Marcus, E., Stona, A. C., Bezborodovs, N., Evans-Lacko, S., . . . Thornicroft, G. (2015). Evidence for effective interventions to reduce mental health-related stigma and discrimination in the medium and long term: Systematic review. *British Journal of Psychiatry, 207*(5), 377-384. doi:10.1192/bjp.bp.114.151944

Thornicroft, G., Mehta, N., Clement, S., Evans-Lacko, S., Doherty, M., Rose, D., . . . Henderson, C. (2016). Evidence for effective interventions to reduce mental-health-related stigma and discrimination. *The Lancet, 387*(10023), 1123-1132. doi:http://dx.doi.org/10.1016/S0140-6736(15)00298-6

Table 1. Trainings with primary health care workers: Intervention content

Reference	Theory	Stigma as a topic	Diagnostic skills	Treatment /interventions /practices	Relationship with patients	mh promotion in communities and psycho-social inter-ventions	policy, regulations, mh system	Stress-management
Abayomi *et al.* (2013)	X			X				
Alexander *et al.* (2013)	X		X					
Armstrong *et al.* (2011)	X	X	X	X		X		
Bradshaw *et al.* (2006)	X			X	X	X		
Chinnayya *et al.* (1990)								
Cui *et al.* (2015)	X		X					X
da Silva Cais *et al.* (2011)	X	X	X	X				
Hofmann-Broussard *et al.* (2017)	X	X	X	X		X		
Li *et al.* (2014a)	X	X	X	X				
Li *et al.* (2015)	X	X					X	
Makanjuola *et al.* (2012)	X		X	X			X	
Motallebi, 1996, included in systematic								

Reference	Theory	Stigma as a topic	Diagnostic skills	Treatment /interventions /practices	Relationship with patients	mh promotion in communities and psycho-social inter-ventions	policy, regulations, mh system	Stress-management
review by Mansouri *et al.* (2009)								
Sadik *et al.* (2011)	X		X	X	X		X	
Shirazi *et al.* (2009)	X		X	X				
Ucok *et al.* (2006)	X	X						
Vesel *et al.* (2015)	X			X	X			X
Villamil-Salcedo *et al.* (2017)	X	X	X	X				
Wang *et al.* (2012)	X		X	X	X			

Table 2. Trainings with primary health care workers: Didactic methods

Reference	Lecture	Interactive methods	Clinical practice / supervision	Recovery story	Role play	Case studies	Handing out written materials	Multimedia
Abayomi *et al.* (2013)								
Alexander *et al.* (2013)	X	X					X	
Armstrong *et al.* (2011)	X	X			X	X		

Reference	Lecture	Interactive methods	Clinical practice / supervision	Recovery story	Role play	Case studies	Handing out written materials	Multimedia
Bradshaw *et al.* (2006)								
Chinnayya *et al.* (1990)	X				X	X		
Cui *et al.* (2015)		X				X		
da Silva Cais *et al.* (2011)	X		X					
Hofmann-Broussard *et al.* (2017)	X			X		X		
Li *et al.* (2014a)	X							
Li *et al.* (2015)	X		X				X	
Makanjuola *et al.* (2012)	X	X			X			X
Motallebi, 1996, included in systematic review by Mansouri *et al.* (2009)								
Sadik *et al.* (2011)	X	X			X		X	X
Shirazi *et al.* (2009)	X	X			X	X		X
Ucok *et al.* (2006)	X					X	X	
Vesel *et al.* (2015)		X						

Reference	Lecture	Interactive methods	Clinical practice / supervision	Recovery story	Role play	Case studies	Handing out written materials	Multimedia
Villamil-Salcedo *et al.* (2017)		X	X			X	X	
Wang *et al.* (2012)	X	X				X		

Table 3. Trainings with primary health care workers: Mental disorders addressed and cultural adaptation of the intervention

Reference	Depression	Schizophrenia	Suicide	Mental disorders in general	Cultural adaptation
Abayomi *et al.* (2013)				X	
Alexander *et al.* (2013)	X				
Armstrong *et al.* (2011)				X	X
Bradshaw *et al.* (2006)				X	X
Chinnayya *et al.* (1990)				X	
Cui *et al.* (2015)				X	
da Silva Cais *et al.* (2011)			X		
Hofmann-Broussard *et al.* (2017)				X	

**Reference**	**Depression**	**Schizophrenia**	**Suicide**	**Mental disorders in general**	**Cultural adaptation**
Li *et al.* (2014a)		X		X	
Li *et al.* (2015)				X	
Makanjuola *et al.* (2012)				X	X
Motallebi, 1996, included in systematic review by Mansouri *et al.* (2009)				X	
Sadik *et al.* (2011)				X	X
Shirazi *et al.* (2009)	X				
Ucok *et al.* (2006)		X			
Vesel *et al.* (2015)				X	
Villamil-Salcedo *et al.* (2017)	X				
Wang *et al.* (2012)	X				X

Risk of bias assessment: Studies with primary health care staff

	Control group	Randomi–zation	Random sequence generation	Allocation concealment	Incomplete outcome data	Comments
Abayomi et al. (2013)	no	n.a.	n.a.	n.a.	high	
Alexander et al. (2013)	yes	yes	uncertain	high	high	waitlist control condition
Armstrong et al. (2011)	no	n.a.	n.a.	n.a.	moderate	
Bradshaw et al. (2006)	n.a.	n.a.	n.a.	n.a.	n.a.	qualitative assessment
Chinnayya et al. (1990)	no	n.a.	n.a.	n.a.	uncertain	
Cui et al. (2015)	no	n.a.	n.a.	n.a.	uncertain	
da Silva Cais et al. (2011)	no	n.a.	n.a.	n.a.	high	
Hofmann-Broussard et al. (2017)	yes	no	n.a.	high	low	quasi-experimental design
Li et al. (2014a)	no	n.a.	n.a.	n.a.	high	
Li et al. (2015)	yes	no	n.a.	n.a.	moderate	quasi-experimental design
Makanjuola et al. (2012)	no	n.a.	n.a.	n.a.	uncertain	
Motallebi (1996)	uncertain	uncertain	uncertain	uncertain	uncertain	results reported in a systematic review
Sadik et al. (2011)	yes	no	n.a.	n.a.	moderate	
Shirazi et al. (2009)	yes	yes	uncertain	low	moderate	
Ucok et al. (2006)	no	n.a.	n.a.	n.a.	high	
Vesel et al. (2015)	yes	no	n.a.	n.a.	uncertain	quasi-experimental design
Villamil-Salcedo et al. (2017)	n.a.	n.a.	n.a.	n.a.	n.a.	qualitative assessment
Wang et al. (2012)	no	n.a.	n.a.	n.a.	high	
